# Factor substitution in Swiss manufacturing: empirical evidence using micro panel data

**DOI:** 10.1186/s41937-017-0016-5

**Published:** 2018-02-20

**Authors:** Sebastian M. Deininger, Lukas Mohler, Daniel Mueller

**Affiliations:** 1 Basel Chamber of Commerce, St. Jakobs-Strasse 25, Basel, 4010 Switzerland; 2 Faculty of Business and Economics, University of Basel, Peter Merian-Weg 6, Basel, 4002 Switzerland; 3 Economist, Secretariat of the Swiss Competition Commission, Hallwylstrasse 4, Bern, 3003 Switzerland

**Keywords:** Substitution elasticities, Complementarity, Swiss manufacturing, Micro panel data, Linear logit, Translog, C33, D24, Q41

## Abstract

This paper analyzes the relationship between factor substitutability and the energy intensity of manufacturing firms. Specifically, we compare the degree of substitutability between the input factors capital, labor, energy, and material for firms with low, medium, and high energy cost shares using a panel of Swiss manufacturing companies covering the period from 1997 to 2008. Our findings indicate substitutability between almost all production factors with one notable exception. Energy and capital are complements in the energy-intensive firm sample: A 1% increase in energy prices decreases capital use by 0.09%. We show that this complementarity is gradually increasing in the energy intensity of firms and draft important policy implications.

## Background

As many other European countries, Switzerland has revised its environmental and energy policy strategies in order to comply with GHG emission reduction goals and foster the transition from fossil fuels to renewable energy^1^. Specifically, Switzerland has decided to reduce GHG emissions by 20% by 2020 compared with 1990 levels. In 2008, a carbon tax was introduced at 12 Swiss francs per metric ton of CO_2_. The tax was raised to 84 Swiss francs in 2016, and a further increase is possible if emissions are above target. Large emitters are exempted from the carbon tax and instead participate in a cap and trade system. Moreover, small- and medium-size companies can also be exempted from the tax provided they commit to legally binding CO_2_ reduction goals.

Policy makers are confronted with the challenge of achieving the environmental targets without negatively affecting the overall economy and the competitiveness of firms. To evaluate the economic impact of such policies, a better understanding of substitution possibilities at the level of individual firms is needed. In this contribution, we analyze the relationship between factor substitutability and the energy intensity of Swiss manufacturing firms^2^. Our panel dataset comprises approximately 7400 observations at the firm level from 1997 to 2008. Using this micro panel data, we estimate substitution elasticities between capital, labor, energy, and material inputs using the linear logit as well as the translog cost function.

Our work is related to the few existing studies that estimate substitution elasticities using firm-level data. Woodland (1993) is the first study to use micro data to analyze substitution between capital, labor, and four energy types in Australia. Nguyen and Streitwieser (1999) examine whether plant size in US manufacturing has an impact on the degree of factor substitution. Arnberg and Bjorner (2007) apply cross-section and panel data techniques to a dataset of Danish firms, and Tamminen and Tuomaala (2012) estimate substitution elasticities for 71 sectors employing a large panel of service and manufacturing firms from Finland. Similar to the last two studies, we employ a panel of manufacturing firms and estimate substitution elasticities controlling for time-invariant unobserved heterogeneity. As a novel contribution to this literature, our focus lies on the relationship between factor substitutability and the energy intensity of manufacturing firms.

To perform our analysis, we classify the firms into three subsets according to their energy intensity and estimate elasticities for these subsets. We stress three main results:

First, we find evidence for substitutability between energy and the input factors capital and labor for firms with low-energy intensity, implying that upon an energy price increase, factor use of labor and capital will increase in order to optimally compensate for the decreasing energy use. In contrast, capital and energy are estimated to be complements for the energy-intensive subset. Specifically, these energy-intensive firms substitute the decreasing energy (− 1.03%) and capital (− 0.09%) use upon an energy price increase of 1% with a higher input of material (+ 0.11%) and, to a lesser extent, labor (+ 0.03%).

Second, by gradually excluding the firms with the lowest energy cost shares from the energy-intensive subset, we are able to show that the higher the mean energy cost share of the remaining firms is, the stronger the complementarity between energy and capital becomes. This result indicates that it is important to account for the heterogeneity of firms when estimating substitution elasticities: Even when using micro panel data, estimating the elasticities over a broad range of firms with heterogeneous characteristics seems to average out the specific substitution behavior of firms at the margin—in our case, the most energy-intensive firms.

Third, these results bear strong policy implications: If energy and capital are complements, higher energy prices lead to lower capital levels, i.e., a reduction in investment. Energy taxes can thus be harmful and negatively affect competitiveness and overall economic performance. As is argued by Tovar and Iglesias (2013), in such a case, it would be beneficial to encourage technological innovation instead of increasing energy prices to achieve reductions in emissions or energy use. Our result thus lends support to policies that exempt certain firms from energy taxes in favor of implementing efficiency measures. In Switzerland, most firms have the choice between paying the carbon tax and committing to reduction goals. There is initial evidence in Oberauner (2010) that, in fact, the higher a firm’s energy intensity is, the more likely it will make use of this possibility. Hence, this may be a policy strategy to prevent the aforementioned harmful effects for energy-intensive firms from realizing. Such a strategy may also be eligible in other countries if evidence for complementarity between energy and capital is identified. As another consequence from a policy perspective, our results hint at possible issues using computable general equilibrium (CGE) models for policy evaluation, since these models often do not take into account the possibility of this kind of complementarity.

The remainder of the paper is organized as follows: the “Related literature” section gives a brief overview and discussion of previous micro data studies in the field of factor substitution. After introducing the applied methodology in the “Methods” section, the empirical analysis is provided in the “Empirical analysis” section. In the “Interpretation of the complementarity result” section, the results are interpreted and policy implications are derived. The paper concludes with the “Conclusions” section.

## Related literature

Research on substitution possibilities between energy and other production factors emerged after the first oil crisis in the 1970s. Earlier studies predominantly estimated substitution elasticities using time series or cross-section data for specific industrial sectors or aggregate manufacturing. Enhanced data availability as well as more sophisticated estimation methods have increased the interest in micro data studies. The empirical literature has shown that elasticity estimates vary substantially and depend on the level of sector aggregation, the geographical region, the time period, and the applied model specification (Koetse et al. 2008).

The majority of studies find that production factors are substitutes in the production process. However, there is an ongoing controversy on whether the factors energy and capital are substitutes or complements. Cross-section studies tend to detect substitutability, while in time series studies, these factors are predominantly found to be complementary (Apostolakis 1990). It is argued that the former measure long-run elasticities, whereas time series capture short-run effects. More recently, such discrepancies have also been observed between cross-section studies and panel studies based on micro panel data. Arnberg and Bjorner (2007) argue that endogeneity problems with labor and energy prices in cross-section studies might cause the differences.

A general issue of studies using aggregated industry data is the difficulty to distinguish between factor substitution and concurrent effects. For instance, Solow (1987) demonstrates convincingly that compositional changes in output can lead to incorrect substitution estimates in studies using aggregate data. He concludes that “[f]actor substitution is a microeconomic phenomenon, and is best examined by looking at microeconomic data” (p. 612). However, only a few micro data studies estimate factor substitution between energy and non-energy factors. The main reason is that energy expenses are rarely available for individual firms. Below, we summarize the results of previous micro data studies in the field of factor substitution.

The first micro data study on substitution between energy and non-energy factors was conducted by Woodland (1993). He used repeated cross-sectional data of approximately 10,000 manufacturing firms in New South Wales, Australia, covering the period from 1977 to 1985. Woodland focused on different types of fuels (coal, oil, gas, and electricity) as well as labor and capital. He found that the demand for energy fuels is price-elastic (with the exception of coal), whereas the demand for capital and labor is price-inelastic. Moreover, he shows that substitution between fuels and the non-energy factors appears to be much stronger than substitution between different types of fuels.

Nguyen and Streitwieser (1999) investigate whether differences exist in factor substitution between small and large production firms. They use cross-sectional data comprising 10,412 US industrial companies in 1991 to estimate the standard KLEM (capital, labor, energy, and material) model applying the translog function. Nguyen and Streitwieserfind that the demand of all four factors is price-elastic, with energy having the highest value and capital the lowest. Furthermore, when considering the Allen-Uzawa elasticity of substitution (AES) and the cross-price elasticity (CPE) as a measure of substitution, they find that the factors capital and energy are either substitutes or complements depending on the size of employment.

Arnberg and Bjorner (2007) apply cross-section and panel data techniques to a dataset of 903 Danish industrial firms for 1993 and the period from 1995 to 1997. They estimate substitution elasticities between the factors electricity, other energy, labor and (machine) capital using the translog and the linear logit function. Their main finding is that, in the fixed-effects model, electricity and capital as well as other energy and capital are complements, whereas they are substitutes in the cross-section model. They point out that the results of the cross-section model might suffer from biased estimates due to endogeneity problems with the price of labor and energy. They argue that firm fixed effects can control for unobservable quality differences among employees, as well as for differences of energy fuels. Similar to other studies, Arnberg and Bjorner find lower values for inter-fuel substitution elasticities than for the elasticities between energy and non-energy factors.

Finally, Tamminen and Tuomaala (2012) employ panel data from 2000 to 2009 comprising 230,000 manufacturing and service companies operating in Finland. They estimate substitution elasticities for the factors labor, capital, outside services, electricity, and other energy forms for 71 sectors. Their results show that the factors labor and capital are relatively price-inelastic. In contrast, material and energy inputs are price-sensitive. Furthermore, as substitution elasticities significantly differ across the 71 sectors, they recommend using sector-specific estimates in CGE models.

## Methods

### Modeling approach

The translog (TL) function introduced by Christensen et al. (1973) is the preferred production function used in the literature because of its functional flexibility and the relatively low data requirements. While the majority of empirical studies make use of the TL function, more recent work also considers the linear logit (LL) function as developed in Considine and Mount (1984) as a functional specification. While the LL function is as flexible as the TL function, it has the advantage that it is well-behaved for a broader range of factor prices and shares. The LL model is especially suitable if some cost shares are small (Considine 1989) and if there is relatively large variation between firms in the cost shares (Arnberg and Bjorner 2007). For these reasons, elasticities are estimated from both the LL and the TL model in order to analyze the substitution possibilities of Swiss manufacturing firms. One contribution of our paper is a direct comparison of results from the LL and the TL models, also checking the concavity constraints in both model types.

#### The linear logit function

We use the logistic production function with the factors capital (K), labor (L), energy (E), and material (M) to represent the production function of firms, developed in Considine and Mount (1984). In the LL model, the factor shares can be represented as

1 $$ s_{in,t}=\frac{\exp\Big(\beta_{in}+\sum\limits_{j}\beta_{ij} \cdot \ln (p_{jn,t})+\beta_{iy} \cdot \ln y_{n,t}\Big)}{\sum\limits_{i}\exp\Big(\beta_{in}+\sum\limits_{j}\beta_{ij} \cdot \ln (p_{jn,t})+\beta_{iy} \cdot \ln y_{n,t}\Big)},  $$

where *i* and *j* stand for the respective input factors (K,L,E,M), *p* and *y* denote the factor price and output of firm *n*, respectively, at time *t*. To estimate the LL model, we linearize it and directly impose the homogeneity and the symmetry restrictions: Following the procedure of Arnberg and Bjorner (2007), we transform the share equations in such a way that the restrictions can be imposed directly into our system of equations, by defining $\beta _{ij}^{\ast }=\beta _{ij}/m_{in}$, where *m*_*in*_ is firm *n*’s mean cost share of input factor *i*. The symmetry restriction implies that $\beta _{ji}^{\ast }=\beta _{ij}^{\ast }$. Homogeneity of degree zero in prices of the production function furthermore implies that $s_{i}\beta _{ii}^{\ast }=d-\sum _{j}^{j\neq i}s_{j} \cdot \beta _{ij}^{\ast }$, where *d* is an unknown scalar. Finally, we have to drop one share equation to obtain a non-singular system. This is done by dividing each share equation by the material share equation and by taking the logarithm. By dividing by the material share equation, the denominator in Eq. (1) cancels out and the logarithm linearizes the functional form. Applying the proposed normalization (*d*=*β*_M*n*_=*β*_M*y*_=0) from Considine and Mount (1984) and adding an error term yields the system of share equations ready for estimation:

2 $$\begin{array}{@{}rcl@{}}  \ln \left(\frac{s_{in,t}}{s_{\textsc{m}n,t}}\right) &=&\beta_{in} + \sum_{j \neq i} \left(\beta_{ji}^{\ast}-\beta_{j\textsc{m}}^{\ast}\right) \cdot m_{jn} \cdot \ln \left(\frac{p_{jn,t}}{p_{\textsc{m}n,t}} \right) \\ && \begin{array}{c} -\left[\sum_{j \neq i} \beta_{ij}^{\ast} \cdot m_{jn} \right]\end{array} \cdot \ln \left(\frac{p_{in,t}}{p_{\textsc{m}n,t}}\right) \notag\\ &&\, - \beta_{i\textsc{m}}^{\ast}\cdot(m_{\textsc{m}n} + m_{in})\cdot \ln\left(\frac{p_{in,t}}{p_{\textsc{m}n,t}}\right) \\ && \,+ \beta_{iy} \cdot \ln y_{n,t} + \varepsilon_{in,t}, \end{array} $$

for *i* and *j* = { K,L,E}. The remaining parameter values can be derived by using the imposed symmetry and homogeneity restrictions.

#### The translog function

The TL function was proposed in Christensen et al. (1973) and has become a popular modeling approach. The main reason for the success of the TL is its flexible functional form which does not impose any prior constraints on the elasticities. Typically, the elasticities of substitution are derived from cost functions^3^. The TL function requires two model restrictions to be fulfilled. First, the factor shares *s*_*in,t*_ have to sum up to 1 at each point in time and for each individual firm *n*. Furthermore, symmetry has to be satisfied, such that *β*_*ij*_=*β*_*ji*_. The factor share equations of the TL cost function can be stated as follows:

3 $$ s_{in,t} = \beta_{in} + \sum_{j=1}^{4} \beta_{ij}\cdot \ln \left(p_{jn,t} \right) + \beta_{iy}\cdot\ln \left(y_{n,t} \right) + \varepsilon_{in,t},  $$

where *i* and *j* denote the four considered factor inputs capital, labor, energy, and material, while *n* and *t* stand for the firm and the time index, respectively, and *p* denotes the factor price. In the right-hand side of the equation, *β*_*in*_ captures input and firm-specific effects which are considered to be constant over time when using panel data. Furthermore, the log of output (*y*) of firm *n* at time *t* is included to control for different production levels. Finally, an error term denoted by *ε*_*in,t*_ is added to the equation.

The adding up restriction of the factor shares leads to singularity because the sum of error terms is zero for each firm. To obtain a non-singular system of equations, we omit the factor share equation for material and normalize the remaining factor share equations by the price of material. The reformulated factor share equation is

4 $$ s_{in,t} = \beta_{in} + \sum_{j=1}^{3} \beta_{ij}\cdot \ln \left(\frac{p_{jn,t}}{p_{\textsc{m}n,t}} \right) + \beta_{iy}\cdot \ln \left(y_{n,t} \right) + \varepsilon_{in,t}.  $$

### Estimation approach

The factor shares of the LL and the TL models (Eqs. (2) and (4)) can best be estimated by using a system of equations approach. Specifically, we refer to a pooled regression approach using seemingly unrelated regression (SUR) on transformed data with firm fixed effects. We transform the data as in Cameron and Trivedi (2009) by calculating the variables’ mean over time for every firm and subsequently performing a within transformation.

Considering the symmetry conditions, the system of equations can be estimated by pooled OLS, or, as in this paper, by the SUR approach which accounts for error correlations across the system of equations. The simultaneous estimation of the model, which is also applied in Arnberg and Bjorner (2007), is more efficient compared to the equation-by-equation OLS estimation and allows for a straightforward implementation of the various parameter restrictions. Furthermore, SUR accounts for cross-equation contemporaneous correlations but assumes cross-time independence of the residual vectors. The SUR estimation considers neither the correlation between individuals (firms) nor the correlation of error terms over time. Consequently, the estimated standard errors are not valid and have to be corrected. We use bootstrap methods to calculate cluster-robust standard errors (Efron 1979).

### Measuring factor substitution

We examine the substitutability of the production factors using the cross-price elasticity of demand (CPE) as a commonly used measure in previous studies^4^.

The CPE between the factors *i* and *j* (*η*_*ij*_) measures the relative change in the quantity of factor *i* (*q*_*i*_) due to a relative change in the price of factor *j* (*p*_*j*_). It is therefore called a *one-factor-one-price* elasticity. Equation (5) presents the elasticity formulas of *η*_*ij*_ and *η*_*ii*_ for the LL model at the point of symmetry.

5 $$ \eta_{ij} = s_{j} \cdot \hat \beta_{ij}^{\ast} + s_{j}, \quad \eta_{ii} = - \sum_{j \neq i} s_{j} \cdot \hat \beta_{ij}^{\ast} + s_{i} - 1 \quad \text{for all \(i,j\),}   $$

where the second term illustrates the special case of an own-price elasticity (OPE)^5^. The elasticities of the factor material can be calculated by using the adding-up and homogeneity conditions. Equation (6) shows the elasticity formulas for the TL model:

6 $$ \eta_{ij} = \frac{\hat \beta_{ij} + s_{i}\cdot s_{j}}{s_{i}}, \quad \eta_{jj} = \frac{\hat \beta_{jj} + s_{j}\cdot s_{j} - s_{j}}{s_{j}}, \quad \text{for all \(i,j\).}   $$

If *η*_*ij*_>0, a price increase of input factor *j* leads to a higher quantity of factor *i*, with output and all other prices held constant. Firms compensate the price increase of factor *j* by using a higher amount of factor *i* instead. Consequently, the input factors are substitutes. If, on the other hand, *η*_*ij*_<0, a price increase in *j* decreases the demand for factor *i*. Thus, firms reduce the amounts of factors *i* and *j* in the production process, to maintain a constant output level. In this case, inputs are considered to be complements.

## Result and discussion

### Empirical analysis

#### Data description and model selection

**Swiss manufacturing data** We use firm-level panel data comprising capital, labor, energy, and material expenditures as input factors for the period from 1997 to 2008. These data as well as the number of employees and the firm’s output are collected in the context of the survey “Production and value added statistics” (WS), conducted by the Swiss Federal Statistical Office (SFSO). The survey levies detailed information on the balance sheets and the income statements of Swiss firms in manufacturing, retail, and services. A total of 10,400 companies were interviewed in 1997/1998, and this number increased to about 20,000 companies in 2008/2009. The response rate is about 90% for large firms, 70% for medium firms, and 60% for small firms. The WS survey has been published since 1997 on an annual basis. The sample used in this study comprises 1965 manufacturing firms (7396 observations) from 22 industry divisions^6^.

Factor cost shares of an input are obtained by dividing the cost of one input factor by the total costs of all considered factors used in production. While this approach is suitable for labor, energy, and material, obtaining the annual real consumption of capital is a challenging task. Arnberg and Bjorner (2007) use machine capital and include the level of building capital as an explanatory variable in the empirical specification, whereas Woodland (1993) calculates the share of capital as a residual value after subtracting the cost of labor, energy, and material from the firm’s output. In this paper, we follow the second approach.

Before calculating the factor shares, we deflate the nominal values of the input factors. To this end, we use a weighted capital deflator and sector-specific deflators for material and output that are taken from the OECD. Sector-specific energy price indices are calculated on the basis of price surveys from the IEA and the SFOE, as well as the survey “Energy consumption statistics in the industry and services sectors” (EVID) published by the SFOE. Because energy price indices are available for 12 aggregate sectors, we combine the 22 industry divisions into 12 manufacturing sectors as displayed in Table 1,^7^. In line with Arnberg and Bjorner (2007), the price index for labor is obtained by dividing the annual wage bill of the company *n* by the number of employees (full-time equivalents). As they mention, the price of labor tends to depend on the quality of labor chosen, implying that endogeneity might be an issue. However, they argue that if labor quality is firm-specific and does not vary over time, fixed effects mitigate the endogeneity problem. This line of reasoning applies analogously to the remaining input factors because the energy mix of demand, capital good requirements, and materials are likely to be firm-specific rather than time-dependent^8^.

**Table 1:** Firms according to industry sector

				Energy cost share		
Sector	Division	Industry description	Obs.	Mean	Q _0.25_	Q _0.75_
1	15, 16	Food products, beverages, and tobacco products	746	2.8	1.0	3.0
2	17–19	Textiles, wearing apparel, leather, and related products	555	2.1	1.0	2.6
3	20	Wood, products of wood, and cork	279	1.7	0.8	2.6
4	21, 22	Paper, paper products, printing, and publishing	914	1.9	0.8	2.0
5	24	Chemicals, chemical, and pharmaceutical products	392	1.9	0.8	2.0
6	25	Rubber and plastic products	471	1.7	0.8	1.8
7	26	Other non-metallic mineral products	317	2.0	0.9	2.3
8	27, 28	Basic metals and fabricated metal products	1256	2.2	0.7	2.6
9	29	Machinery and equipment n.e.c.	790	1.9	0.6	2.2
10	30–33	Electrical equipment, electronic, and optical products	1152	1.6	0.8	1.7
11	34, 35	Motor vehicles and other transport equipment	186	1.8	0.8	2.2
12	36, 37	Furniture and other manufacturing	338	2.2	1.0	2.7

Notes: Divisions according to NOGA 2002 industrial classification of Switzerland, two-digit. Mean factor shares and quantiles are denoted in percent. *n.e.c.* not elsewhere classified

**Fig. 1:**
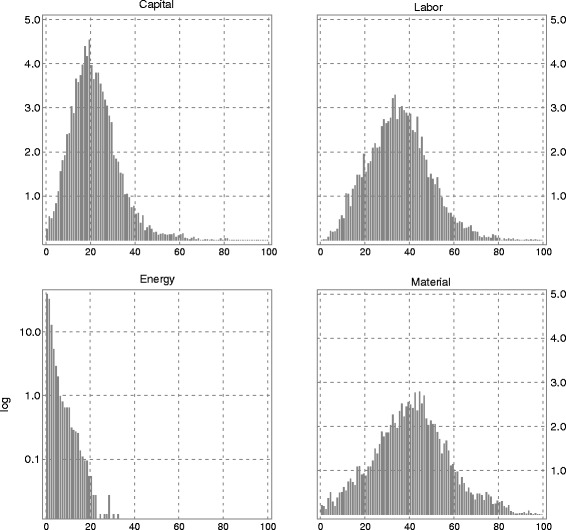
Histogram of factor cost shares across firms. Note: The histograms show the frequency of observations (*y*-axis) from 1997 to 2008 by the share of the respective factor (capital, labor, energy, and material) within the full sample (*x*-axis) in percent

**Factor cost shares and firm subsets** In the production process of Swiss manufacturing firms, the factor with the largest mean cost share over the observed period is material (41.0%), followed by labor (35.5%), capital (21.4%), and energy (2.0%). The mean cost share of energy in Switzerland is about half the cost share observed for US manufacturing firms in the dataset of Nguyen and Streitwieser (1999). The energy cost share (electricity and other energy) of the Danish firm sample is roughly 4.5% of aggregate costs (Arnberg and Bjorner 2007)^9^. As can be seen from Fig. 1, the firms’ mean cost shares of capital, labor, and material exhibit a bell-shaped distribution. In marked contrast, the firms’ mean energy cost shares are highly skewed to the right (even if presented on a log scaled *y*-axis): Half of the firms in our sample have mean energy cost shares of less than the median value of 1.4%. A few firms, however, feature mean energy cost shares of 20% or more.

Given these striking differences between firms with regard to their energy cost shares, we aim to analyze factor substitution possibilities between input factors taking into account different levels of energy needs in the firms’ production processes. Higher energy prices will hit the companies which have high energy shares hardest. However, firms that can easily substitute energy using other production factors are able to mitigate the effects of rising energy prices to a greater extent. One strategy that comes to mind to analyze these possible differences is to split the sample into subsets according to the average energy cost share of the predetermined industry sectors as shown in Table 1. As it turns out, however, the heterogeneity within these sectors regarding the energy cost share is large and at the same time, the heterogeneity between the sectors is relatively small: As the last three columns of the table show, sectors do not differ much regarding the average energy cost share, and in each sector, there are firms with high and with low energy cost shares^10^. Hence, we cannot follow this strategy in our analysis^11^.

Our approach is to allocate each firm to one of three approximately equally sized subsets according to the firm’s mean energy cost share over the whole observation period^12^. The first subset comprises a third of all firms, namely those with the smallest mean energy cost share (low energy-use firms, below 1.1%). The second subset consists of another third of all firms, namely those with medium mean energy cost shares (medium energy-use firms, between 1.1 and 2.0%). Subset 3 contains the remaining third of firms, namely those with the highest mean energy cost share (high energy-use firms, above 2.0%). Summary statistics on the three subsets can be found in Table 2. While capital, labor, and material shares are of similar magnitude in all three subsets, the high energy-use subset exhibits a mean energy cost share of 4.0%, more than six times higher than the mean energy cost share in the low energy-use subset (0.6%). The allocation of the firms into three categories of equal size is, to a large extent, arbitrary. Furthermore, it is performed by using an endogenously determined variable, namely the energy cost share. This approach therefore relies on certain assumptions that may influence our results. We analyze the impact of these assumptions in the “Sensitivity of the results” section.

**Table 2:** Mean factor shares of firm subsets

Energy use	All	Low	Medium	High
Mean capital share	21.4	22.6	20.2	21.5
Mean labor share	35.5	34.1	36.1	36.3
Mean energy share	2.0	0.6	1.4	4.0
Mean material share	41.0	42.7	42.2	38.2
Observations	7396	2462	2461	2473

Notes: Mean factor shares are denoted in percent

**Specification tests and model selection** Before discussing the main results using the three subsets and the full sample, we perform different statistical tests in order to motivate our modeling strategy and make sure that important requirements are met. We first check whether the Cobb-Douglas function would be sufficient to fit the production process of Swiss manufacturing firms. This production function assumes the absence of cross-substitution possibilities between factors. Tests for the hypothesis that cross-substitution possibilities can be disregarded and the Cobb-Douglas function is adequate are rejected for our three subsets^13^. Thus, we do need the more complex LL or TL models to map the production processes in Swiss manufacturing.

Second, we test whether the cost function is concave at the sample means and at each observation when using the LL or TL model because the concavity condition is not globally satisfied. Concavity violations may indicate a misspecification of the underlying production model and result in biased elasticity estimates (Diewert and Wales 1987). Table 12 in the “Appendix” section displays the eigenvalues of the Hessian matrix at the sample means and the percentage of observations that satisfy concavity for both modeling approaches, all four samples and different parametrizations of the model^14^. The table indicates that concavity at the sample means is satisfied for all specifications of the LL model and most specifications of the TL model, since all eigenvalues are negative semi-definite. Checking the percentage of concave observations, we find more diverse results. The specification we will use in the empirical analysis for each modeling approach and each of the four samples is shaded in gray: Using the LL model, 100% of the concavity restrictions are met for the full sample as well as the low and medium energy-use subsets. For the high energy-use subset, 90% of all concavity restrictions are met. The TL model achieves 96.5% in the high energy-use sample and hence performs slightly better than the LL model^15^. Using the other three samples, however, the TL model performs slightly worse than the LL model. Considering the good performance of both modeling approaches with respect to concavity, we present the results from both specifications in the next section.

#### Main results

In the following, we present the OPEs and CPEs using the LL and the TL modeling approaches. As the elasticity estimates under the two models are in most cases close to each other but the standard errors are usually lower in the LL model, we focus on the LL results in the following discussion and refer to the “Sensitivity of the results” section for a comparison of the results of the two functional specifications. The estimated parameter values from the systems of equations used to derive the elasticities are displayed in Table 13 in the “Appendix” section.

In our description of the results, we focus on the firms’ reaction upon an energy price change. Remember that, by definition, the estimated elasticities describe firms’ optimal adjustments to their production processes under the assumption of constant output quantity. If increased energy prices lead to a decrease in energy use in the production process, one or more of the other factors have to increase to hold the outputs of firms at a constant level. Hence, by construction there will always be at least one of the other factors that is substitutable with energy. The other two factors are substitutes or complements vis-a-vis the factor energy.

**Full sample** Table 3 displays the OPEs of the sample containing all available firms. Estimates from the LL specification are displayed in the left panel, and estimates from the TL specification in the right panel. The elasticity estimates of both specifications are supplemented by a graphical representation of the 95% confidence intervals. Using the full sample, all OPEs are negative and significantly different from zero. The OPE of capital is close to one; labor, energy—with a particularly large standard error—and material are slightly less price-elastic.

**Table 3:** Own-price elasticities from the full sample

	

Notes: The symbols ^∗^, ^∗∗^, and ^∗∗∗^ denote significance at the 10, 5, and 1% levels, respectively. The figures show 95% confidence intervals for the respective parameter estimate

The CPEs are presented in Table 4. The first three elasticities— *η*_*LK*_, *η*_*EK*_, and *η*_*MK*_—measure the relative impact that a price change in the factor capital has on the use of the other three production factors. This is followed by nine elasticities expressing the effect of price changes in labor, energy, and material on the other three factors, respectively. All CPEs are positive and, with the exception of the pair capital and energy, significantly different from zero, indicating substitutability between the production factors. The magnitude of the effect is partly determined by the average factor shares: Adjustments in the price of capital (avg. factor share of 21.4%), labor (35.5%), and material (41.0%) have a larger effect on the use of the other factors than energy (2.0%) price adjustments. Considering an increase in energy prices of 1%, we find the increases in labor (0.007%), material (0.013%), and capital (0.010%, not significantly different from zero) to be small in magnitude.

**Table 4:** Cross-price elasticities from the full sample

	

Notes: The symbols ^∗^, ^∗∗^, and ^∗∗∗^ denote significance at the 10, 5, and 1% levels, respectively. The figures show 95% confidence intervals for the respective parameter estimate

**Low energy-use firm sample** Table 5 displays the estimated OPEs for low energy-use firms with energy shares below 1.1%. The OPE of energy is about − 1, indicating that energy is unit-elastic in demand. The OPEs of the other factors are between − 1 and 0, indicating that the factors capital, labor, and material are slightly less price-sensitive than the factor energy. However, the confidence intervals are relatively wide. All four OPEs are significantly smaller than zero at least at the 5% level.

**Table 5:** Own-price elasticities from the low energy-use firm sample

	

Notes: The symbols ^∗^, ^∗∗^, and ^∗∗∗^ denote significance at the 10, 5, and 1% levels, respectively. The figures show 95% confidence intervals for the respective parameter estimate

Table 6 presents the CPEs of low energy-use firms. All elasticity estimates but one are positive and most are significantly different from zero, including those for the factor pair energy and capital. Given the relatively large standard errors, CPEs do not differ greatly from those estimated from the full sample. Regarding changes in energy prices, we observe that the factors labor (CPE of 0.004), capital (0.020), and material (0.000, not significantly different from zero) are weak substitutes. Again, the small magnitude does not come as a surprise, since mean energy shares are lowest for this group of firms (0.6%).

**Table 6:** Cross-price elasticities from the low energy-use firm sample

	

Notes: The symbols ^∗^, ^∗∗^, and ^∗∗∗^ denote significance at the 10, 5, and 1% levels, respectively. The figures show 95% confidence intervals for the respective parameter estimate

#### Medium energy-use firm sample

Tables 7 and 8 display the estimated OPEs and CPEs for medium energy-use firms. The OPE of capital is lower than − 1, while the OPEs of the other factors are between − 1 and 0. The OPE of energy is not significantly different from zero. Energy price increases have a small and insignificant effect on capital (CPE of 0.022), labor (0.002) and material (0.003).

**Table 7:** Own-price elasticities from the medium energy-use firm sample

	

Notes: The symbols ^∗^, ^∗∗^, and ^∗∗∗^ denote significance at the 10, 5, and 1% levels, respectively. The figures show 95% confidence intervals for the respective parameter estimate

**Table 8:** Cross-price elasticities from the medium energy-use firm sample

	

Notes: The symbols ^∗^, ^∗∗^, and ^∗∗∗^ denote significance at the 10, 5, and 1% levels, respectively. The figures show 95% confidence intervals for the respective parameter estimate

**High energy-use firm sample** Tables 9 and 10 display the OPEs and the CPEs for the high energy-use subset. All estimated OPEs are negative and significantly different from zero at the 1% level, except for the factor energy which is significant at the 10% level. The factors energy and capital are unit-elastic in demand (*η*_EE_=− 1.03, *η*_KK_=−1.09), while the factors labor and material are both inelastic. Again standard errors are relatively high.

**Table 9:** Own-price elasticities from the high energy-use firm sample

	

Notes: The symbols ^∗^, ^∗∗^, ^∗∗∗^ denote significance at the 10, 5, and 1% levels, respectively. The figures show 95% confidence intervals for the respective parameter estimate

**Table 10:** Cross-price elasticities from the high energy-use firm sample

	

Notes: The symbols ^∗^, ^∗∗^, ^∗∗∗^ denote significance at the 10, 5 and 1% levels, respectively. The figures show 95% confidence intervals for the respective parameter estimate

Most CPEs estimated from the high energy-use subset are similar to those shown for firms with low and medium energy use. One exception is the factor pair energy and capital. These input factors are estimated as being complements instead of substitutes in the production process of energy-intensive firms (*η*_KE_=− 0.09,*η*_EK_=− 0.59). The respective elasticities are significantly different from zero at the 10% level using the LL specification (not significant using the TL specification; see the “Sensitivity of the results” and “Sensitivity of the results” sections for a further analysis). If the price of capital increases by 1%, firms reduce the amount of energy by 0.59%. Also, an energy price increase of one percent leads to a decrease in the amount of capital of 0.09%. The magnitude of this effect is about four to five times higher than in the other two subsets and the full sample. Given the still relatively low mean energy cost share of 4.0%, this magnitude is considerable^16^. Before interpreting the results and formulating policy implications, we perform a sensitivity analysis in the next section, focusing mainly on the complementarity between energy and capital for energy-intensive firms, i.e., on the *complementarity result*.

#### Sensitivity of the results

**Fig. 2:**
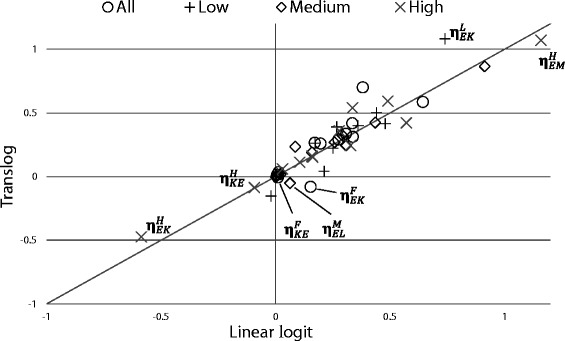
Linear logit versus translog: cross-price elasticity estimates

**Linear logit versus translog modeling** As shown in the “Data description and model selection” section, both modeling approaches satisfy concavity restrictions well. It is unclear, however, how sensitive the estimated elasticities are with respect to the model specification chosen. Figure 2 displays the CPEs of the LL and the TL. The LL results are depicted on the horizontal axis, and the TL results on the vertical axis. If the point estimate of an elasticity is identical for the two specifications, it lies on the 45° line. Overall, most elasticity estimates are close to this line.

A few estimates marked in the figure are worth discussing. First, the negative CPEs between capital and energy in the high energy-use subsets, $\eta _{EK}^{H}$ and $\eta _{KE}^{H}$, are of similar magnitude. Second, there are three estimates with opposite signs, namely $\eta _{EK}^{F}$, $\eta _{KE}^{F}$, and $\eta _{EL}^{M}$. However, these estimates are accompanied by large standard errors and are not significantly different from zero under both modeling approaches. Third, we observe that the elasticity estimates using the full sample (depicted as circles) possess less variation than the three subsets—especially regarding the energy elasticities, for example $\eta _{EK}^{H}$, $\eta _{KE}^{H}$, $\eta _{EM}^{H}$, and $\eta _{EK}^{L}$. This is a first indication that estimating elasticities using data on very heterogeneous firms “averages out” adjustment behavior of the firms at the margin of the distribution. The most striking example of this “averaging out” result is the relationship between the production factors energy and capital: In the low energy-use subset, we find substitutability between the factors energy and capital at the 1% level of significance, and in the high energy-use subset complementarity at the 10% level. Using all firms for the estimation, the relationship becomes insignificant.

The similar results from the LL and the TL models are a first robustness check of the complementarity result: As mentioned, for example, by Considine and Mount (1984) and Arnberg and Bjorner (2007), heterogeneity of the observations regarding the factor shares can be an issue especially when estimating using a translog function. In our case, this does not seem to be a problem.

While the point estimates between the LL and the TL specifications are of similar magnitude, we find that the LL estimates are—as *t*-values are higher on average—more precise than the TL counterparts. An inspection of Tables 4, 6, 8, and 10 shows that under the LL specification, a higher percentage of elasticities are estimated to be significantly different from zero. At the significance level of 10%, for example, 83% of all CPEs are significant both in the full sample (TL 75%) and in the low energy-use subset (TL 50%), while 50% in the medium energy-use subset (TL 50%) and 100% in the high energy-use subset (TL 50%). Since both models satisfy concavity restrictions well, we prefer the LL estimates due to the higher precision.

**Complementarity-concavity tradeoff** In this section, we test whether our finding of complementarity between capital and energy in the production process of energy-intensive firms depends on our definition of the high energy-use subset. Specifically, we gradually exclude the least energy-intensive firms from the sample and re-estimate the two production models with the remaining firms.

Table 11 depicts the cross-price elasticities (*η*_*KE*_,*η*_*EK*_) and the concavity measure for the LL model and the TL model. In addition, the mean factor shares for the used subset are displayed. The original specification as shown in the “Medium energy-use firm sample” section is shaded in gray. Excluding the least energy-intensive firms from the high energy-use subset tends to strengthen the complementarity result in both modeling specifications: The magnitude of the elasticities increases as well as the *t*-values. Using the top 20% of energy-intensive firms, the CPE of an energy price change on capital use changes from − 0.09 to − 0.21 (TL: from − 0.09 to − 0.17) and the CPE of a capital price change on energy use changes from − 0.59 to − 1.03 (TL: from − 0.47 to − 0.76). The results are now significantly different from zero at the 1% level in the LL specification and at the 5% level in the TL specification. This analysis further builds our confidence in the complementarity result. Moreover, this exercise provides additional evidence for an “averaging out” of the elasticity estimates the more heterogeneous the subset is regarding energy intensity.

**Table 11:** Exploring the complementarity result between energy and capital

	

Notes: The table displays the degree of complementarity between capital and energy and the rate of observations that satisfy concavity as well as mean factor shares for different energy intensity subsets. Asterisk denotes whether concavity is satisfied at the respective sample means. Mean factor shares are denoted in percent. The gray-shaded row denotes the high energy use sample used in the sections above

Excluding the least energy-intensive firms and thus reducing the observations available for the estimation comes at a cost: The percentage of observations for which the concavity restriction is satisfied drops gradually, and in some specifications the concavity restriction is not satisfied even at the sample means. Especially in the last two specifications of the LL model (15 and 10%) and the last specification of the TL model (10%), there is ample evidence for model misspecification.

Another interesting observation is revealed by considering the mean factor cost shares. They indicate that the share of capital increases from 21.6 to 24.2% when we exclude the less energy-intensive firms from the high energy-use subset. The production of highly energy-intensive firms tends to be more capital-intensive and less labor- and material-intensive. This link between capital and energy in the production process may be the underlying reason for the complementarity that we find in our analysis.

**Complementarity and subset allocation** As described in the “Data description and model selection” section, we allocate firms to the three subsets by their mean energy cost share. The energy cost share is endogenous to our modeling approach. It would be preferable to split the firm sample by applying a truly exogenous variable to prevent any endogeneity issues. As argued before, given the available data as well as other restrictions, we are not able to use a completely predetermined variable to split our sample and at the same time analyze substitution possibilities of firms with different energy intensity in their production processes. In this section, we examine the sensitivity of our results using different approaches of sample splitting.

We allocate firms to subsets depending on their energy cost share in the first year we observe them^17^. The energy cost share for the first year is not completely exogenous to the analysis, since firms’ previous decisions may also play a role in their subsequent behavior. However, the energy cost share for the first year may be seen as being more predetermined to the analysis than in subsequent years. Results are close to those in the original specification. Specifically, *η*_*KE*_ of the high energy-use subset changes from − 0.093 to − 0.098 (TL: − 0.086 to − 0.088) and *η*_*EK*_ from − 0.587 to − 0.628 (TL: − 0.474 to − 0.493), both still being statistically significant at the 10% level (TL: still insignificant). Repeating the exercise performed in the “Sensitivity of the results” section, i.e., gradually excluding the least energy-intensive firms from the high energy-use subset, again strengthens the complementarity result: Using the top 20% of energy-intensive firms, *η*_*KE*_ increases to − 0.207, *η*_*EK*_ to − 1.034, now statistically significant at the 5% level (TL: − 0.175; − 0.765, now statistically significant at the 10% level). A very similar result is attained if the firms’ last year’s energy cost share is used^18^. Our interpretation of this sensitivity analysis is that the complementarity result is robust and does not depend on the specifics of the subset allocation mechanism.

### Interpretation of the complementarity result

Energy-intensive firms react to an energy price increase of one percent by reducing energy use by 1.03%. At the same time, capital use decreases by 0.09%. This adjustment in capital equipment is substantial, given that the mean capital share of energy-intensive firms is more than five times larger than the mean energy share. To hold output constant, these firms increase material and labor use by 0.11 and 0.03%, respectively. Thus, energy-intensive firms alter their production processes considerably upon energy price increases. As shown in the “Sensitivity of the results” section, this adjustment process will be more pronounced, the higher the energy intensity of firms and thus the stronger the complementarity between energy and capital inputs^19^.

The analysis of the exact forces that are behind this adjustment process of energy-intensive firms in Switzerland is beyond the scope of our contribution. An example may, however, make the interpretation of our results clearer. It is conceivable that energy-intensive firms already produce quite energy-efficiently, since energy represents an important cost factor. Investments in innovations and new technologies that may be necessary upon further energy price increases may be difficult or expensive to implement. Consequently, production is becoming more labor- and material-intensive as various energy- and capital-intensive steps in the production process are replaced. In the context of firms that face international competition, as is common in a small open economy like Switzerland, the energy- and capital-intensive steps may be relocated abroad. In the context of Switzerland’s climate policy and thus concentrating on emissions that are an important part of firms’ energy use, these firms may be exposed to the risk of carbon leakage—the reallocation of energy-intensive production processes to countries with laxer environmental policies^20^.

Irrespective of the true underlying causes, complementarity between energy and capital means that divestment will take place upon an energy price increase. The cessation of investment will affect the growth rates of energy-intensive industries and the overall economy—especially when it is associated with a weakening in the competitiveness of local firms. This argument is in line with Tovar and Iglesias (2013) who warn of the harmful effects of policies that increase energy prices if there is evidence of complementarity between energy and capital. They recommend that, in such a case, instead of applying pricing policies, policy makers should devise policies that promote innovations within these sectors or support energy-efficient technologies. In contrast, pricing policies are advisable if substitutability predominates. Higher energy prices then lead to an increase in other inputs and may also spur investment in energy-efficient technologies.

With these results in mind, the present design of Switzerland’s environmental policy is of interest: With 84 Swiss francs per metric ton, a substantial tax on CO_2_ emissions is effective. However, large emitters are exempted from the tax and instead participate in a carbon emission trading system. These firms receive fewer emission certificates compared to their historical usage and thus have to reduce their emissions or purchase further certificates. Additionally, small and medium firms can avoid being taxed by committing to reduction goals. Oberauner (2010) shows in an empirical contribution that energy-intensive firms use this possibility and commit to efficiency measures instead of paying the tax more often than other firms. Our result, which is based on data from a period before the current environmental policy was implemented, may indicate one reason for this behavior: Differences in the ease of substitution for the input factor energy induce energy-intensive firms to choose a different policy instrument. Given the possible harmful effects of pricing instruments in the presence of complementarity, our results lend support to policies that exempt certain firms from energy taxes being used in combination with efficiency commitments.

## Conclusions

This contribution provides an analysis of factor substitution among capital, labor, energy, and material in Swiss manufacturing using micro panel data at the firm level from 1997 to 2008. We focus on examining the relationship between factor substitutability and the energy intensity of manufacturing firms in Switzerland. To this end, firms are divided into three subsets according to their energy intensity. We find that substitutability between the considered input factors prevails most of the time and that differences between the subsets are not substantial. One notable exception, however, is the complementarity between energy and capital inputs that we find for energy-intensive firms. We subject this result to various sensitivity tests and find that it holds under different modeling strategies as well as different definitions of the high energy-use subset. By gradually excluding the least energy-intensive firms, we furthermore show that the complementarity result strengthens as the mean energy intensity of the remaining firms increases.

We stress important policy implications, namely that in the presence of complementarity between energy and capital inputs, policy measures to reduce energy-use should not aim at introducing energy price increases. Due to the complementarity, such increases may negatively affect firms’ investment decisions and therefore have harmful effects on the competitiveness of firms and overall economic performance. In view of these problems, the encouragement of technological diffusion or the support of technical innovation may be more promising approaches. However, we stress that these latter policies depend on the specific production processes of the affected firms as well as on the specific implementation of the policies. In order to successfully implement and operate such policies, more information is needed than can be given within the scope of our contribution. The exploration of these alternative possibilities is an area for future research.

In a similar vein, the complementarity result is relevant for CGE models that use elasticity estimates as an important input for forecasting and policy analysis. As CGE models often use combinations of Cobb-Douglas and constant elasticity of substitution (CES) production functions, the degree of complementarity that these modeling approaches can account for is naturally restricted. Results from CGE models may therefore be biased for certain energy-intensive firm clusters or industries, and an evaluation of energy pricing policies using these models may be unduly positive.

In the light of existing policy regimes, our results back policies that exempt certain firms from energy or emissions taxes and instead require them to adopt efficiency measures. A possible implementation of a carbon tax which satisfies these requirements can be found in Switzerland: The Swiss system gives most firms the opportunity to evade the GHG emissions tax and instead commit to legally binding CO_2_ reduction goals or participate in a cap and trade system. There is first evidence which confirms that primarily energy-intensive firms make use of this opportunity. Hence, it may be the case that such a flexible system prevents the mentioned possible harmful effects of pricing policies in Switzerland. Moreover, this approach might provide firms with incentives to innovate and reduce emissions, which has been empirically questioned for current policy instruments such as temporary compensation schemes (Antimiani et al. 2016). A flexible system of this kind may also be eligible for other countries. That said, more research is certainly required to identify and analyze the specific mechanisms that are at work. Here, the principle research questions that need to be established are whether a policy that permits exceptions from an emissions tax will suffice to attain reduction targets, to what extent carbon leakage can be prevented, and how energy-intensive firms perform in international competition under such a policy regime.

## Appendix

**Table 12:** Concavity in the translog and the linear logit model

	

Notes: The gray-shaded areas indicate our specification choice based on LR-tests for the linear logit model and the translog model

**Table 13:** Estimated parameter values from the systems of equations

Cost function	Linear logit	Translog						
Energy-use	All	Low	Medium	High	All	Low	Medium	High
*β*_kk_	$\underset {(1.0271)}{-1.5323}^{}$	$\underset {(1.40383)}{-\thinspace 0.3261}$	$\underset {(1.37144)}{-\thinspace 2.8252}^{**}$	$\underset {(1.89095)}{-\thinspace 1.4632}^{}$	$\underset {(0.0253)}{-\thinspace 0.0469}^{*}$	$\underset {(0.04405)}{-\thinspace 0.0146}^{}$	$\underset {(0.03973)}{-\thinspace 0.0859}^{**}$	$\underset {(0.04697)}{-\thinspace 0.0325}^{}$
*β*_kl_	$\underset {(0.1451)}{0.0361}^{}$	$\underset {(0.22205)}{-\thinspace 0.1439}^{}$	$\underset {(0.24317)}{-\thinspace 0.1153}^{}$	$\underset {(0.28905)}{ 0.4746}^{*}$	$\underset {(0.009)}{0.0144}^{}$	$\underset {(0.01667)}{ 0.0115}^{}$	$\underset {(0.01671)}{-\thinspace 0.0062}^{}$	$\underset {(0.02104)}{ 0.0510}^{**}$
*β*_ke_	$\underset {(0.9812)}{-\thinspace 0.1921}^{}$	$\underset {(1.30739)}{ 2.6823}^{**}$	$\underset {(1.26284)}{ 0.7123}^{}$	$\underset {(1.8406)}{-4.1125}^{**}$	$\underset {(0.0064)}{-\thinspace 0.006}^{}$	$\underset {(0.00446)}{ 0.0054}^{}$	$\underset {(0.00428)}{ 0.0005}^{}$	$\underset {(0.01588)}{-\thinspace 0.0278}^{*}$
*β*_km_	$\underset {(0.3294)}{0.7777}^{**}$	$\underset {(0.53995)}{ 0.2512}^{}$	$\underset {(0.4757)}{ 1.4241}^{***}$	$\underset {(0.76975)}{ 0.7326}^{}$	$\underset {(0.0310)}{0.0385}^{}$	$\underset {(0.05161)}{-\thinspace 0.0023}^{}$	$\underset {(0.04804)}{ 0.0916}^{*}$	$\underset {(0.06072)}{ 0.0093}^{}$
*β*_ll_	$\underset {(0.2293)}{0.1659}^{}$	$\underset {(0.34239)}{-\thinspace 0.0876}^{}$	$\underset {(0.3597)}{ 0.3397}^{}$	$\underset {(0.46448)}{ 0.2412}^{}$	$\underset {(0.011)}{0.0129}^{}$	$\underset {(0.0183)}{-\thinspace 0.0355}^{*}$	$\underset {(0.01913)}{ 0.0499}^{***}$	$\underset {(0.01845)}{ 0.0209}^{}$
*β*_le_	$\underset {(0.2302)}{-\thinspace 0.4714}^{**}$	$\underset {(0.34582)}{-\thinspace 0.3215}^{}$	$\underset {(0.35269)}{-\thinspace 0.8072}^{**}$	$\underset {(0.4639)}{ 0.0108}^{}$	$\underset {(0.0021)}{-0018}^{}$	$\underset {(0.00096)}{-\thinspace 0.0019}^{*}$	$\underset {(0.00174)}{-\thinspace 0.0057}^{***}$	$\underset {(0.00629)}{ 0.0072}^{}$
*β*_lm_	$\underset {(0.1030)}{-\thinspace 0.1507}^{}$	$\underset {(0.1605)}{ 0.1518}^{}$	$\underset {(0.16274)}{-\thinspace 0.2123}^{}$	$\underset {(0.20774)}{-\thinspace 0.5160}^{**}$	$\underset {(0.0161)}{-\thinspace 0.0253}^{}$	$\underset {(0.02718)}{ 0.0259}^{}$	$\underset {(0.02806)}{-\thinspace 0.0380}^{}$	$\underset {(0.03152)}{-\thinspace 0.0791}^{**}$
*β*_ee_	$\underset {(0.8534)}{1.8319}^{**}$	$\underset {(1.12522)}{-25.0307}^{***}$	$\underset {(1.09236)}{ 13.6234}^{***}$	$\underset {(1.52994)}{-\thinspace 1.8234}^{}$	$\underset {(0.0054)}{0.0019}^{}$	$\underset {(0.00268)}{ 0.0001}^{}$	$\underset {(0.00362)}{ 0.0077}^{**}$	$\underset {(0.01391)}{-\thinspace 0.0071}^{}$
*β*_em_	$\underset {(0.4231)}{0.0528}^{}$	$\underset {(0.6342)}{-\thinspace 1.0517}^{*}$	$\underset {(0.53985)}{-\thinspace 0.7720}^{}$	$\underset {(0.95967)}{ 2.5192}^{***}$	$\underset {(0.011)}{0.0059}^{}$	$\underset {(0.00699)}{-\thinspace 0.0036}^{}$	$\underset {(0.00775)}{-\thinspace 0.0026}^{}$	$\underset {(0.02741)}{ 0.0277}^{}$
*β*_mm_	$\underset {(0.5460)}{-\thinspace 0.2741}^{}$	$\underset {(0.85543)}{-\thinspace 0.2413}^{}$	$\underset {(0.74858)}{-\thinspace 0.4695}^{}$	$\underset {(1.2455)}{-\thinspace 0.1279}^{}$	$\underset {(0.0367)}{-\thinspace 0.0188}^{}$	$\underset {(0.05867)}{-\thinspace 0.0200}^{}$	$\underset {(0.05643)}{-\thinspace 0.0511}^{}$	$\underset {(0.07306)}{ 0.0421}^{}$
linear trend	$\underset {(0.0026)}{-\thinspace 0.0087}^{***}$		$\underset {(0.00536)}{-\thinspace 0.0136}^{**}$	$\underset {(1e-04)}{-\thinspace 0.0004}^{***}$		$\underset {(0.00417)}{-\thinspace 0.0109}^{***}$		
quadratic trend						$\underset {(1e-05)}{ 3e-05}^{***}$		
*β*_*Ky*_	$\underset {(0.0200)}{-\thinspace 0.0912}^{***}$	$\underset {(0.0407)}{ 0.0010}^{}$	$\underset {(0.02987)}{-\thinspace 0.1820}^{***}$	$\underset {(0.03257)}{-\thinspace 0.0660}^{** }$	$\underset {(0.0019)}{0.0122}^{***}$	$\underset {(0.00299)}{ 0.0175}^{***}$	$\underset {(0.00317)}{ 0.0028}^{}$	$\underset {(0.00334)}{ 0.0182}^{***}$
*β*_*Ly*_	$\underset {(0.0159)}{-\thinspace 0.3492}^{***}$	$\underset {(0.03541)}{-\thinspace 0.3025}^{***}$	$\underset {(0.02483)}{-\thinspace 0.3755}^{***}$	$\underset {(0.02521)}{-\thinspace 0.3589}^{***}$	$\underset {(0.0019)}{-\thinspace 0.0610}^{***}$	$\underset {(0.00382)}{-\thinspace 0.0550}^{***}$	$\underset {(0.00369)}{-\thinspace 0.0629}^{***}$	$\underset {(0.00327)}{-\thinspace 0.0635}^{***}$
*β*_*Ey*_	$\underset {(0.0224)}{-\thinspace 0.2394}^{***}$	$\underset {(0.04123)}{-\thinspace 0.2746}^{***}$	$\underset {(0.02563)}{-\thinspace 0.2315}^{***}$	$\underset {(0.04538)}{-\thinspace 0.2133}^{***}$	$\underset {(0.0004)}{-\thinspace 0.0004}^{}$	$\underset {(0.00021)}{-\thinspace 0.001}^{***}$	$\underset {(0.00027)}{-\thinspace 0.0005}^{*}$	$\underset {(0.00116)}{ 0.0002}^{}$
*N*	7396	2462	2461	2473	7396	2462	2461	2473

Notes: The symbols ^∗^, ^∗∗^, ^∗∗∗^ denote significance at the 10, 5, and 1% levels, respectively. Note that the model restriction requires *β*_*ji*_=*β*_*ij*_. Based on our specification choice presented in Table 12, linear and quadratic trends over time have been included for certain subsets. Cluster-robust standard errors using bootstrap with 5000 replications are presented in parentheses

## Footnotes

1 See OECD (2015) for an in-depth analysis of energy policies in OECD countries and Ekins and Speck (2011) on environmental tax reforms in Europe.

2 For Switzerland, substitution elasticities have been estimated on a sectoral level (Mohler and Mueller 2012).

3 An exception is Berndt and Christensen (1973) who estimate the elasticities directly from the production function.

4 Morishima elasticities of substitution (MES) represent another way of analyzing substitution possibilities. Since they are less commonly used in the literature, we provide a definition of the MES as well as the resulting elasticity estimates in an online appendix.

5 The standard errors of the estimated elasticities can be calculated by using the Delta method (see e.g., Greene (2000)).

6 Only observations of firms stating their energy consumption have been considered. Expenditures on energy are not available by energy carriers. The sample is an unbalanced panel due to firms’ entry, exit, and non-response to the survey. The survey was continued after 2008; however, from 2009 onwards, no information on energy costs of firms has been collected.

7 More precisely, we calculate a chain Laspeyres index where the weights are updated annually by using the expenditure shares of the different energy sources as weights. Sector-specific expenditure shares were computed by using energy prices and physical quantities (measured in TJ) of the major energy sources (electricity, natural gas, light fuel oil, heavy fuel oil, and coal).

8 We checked for possible endogeneity issues by applying 3SLS estimations on data from 1998 to 2008, using the one-period lagged price series. The results are similar to those of the SUR approach.

9 Note that Arnberg and Bjorner (2007) exclude material, which leads to a higher energy share. Excluding material in our sample results in a mean energy cost share of 3.5%.

10 At this level of aggregation, industries are defined very broadly. For example, sector 5 consists of chemical companies with highly energy-intensive physical production processes and pharmaceutical companies which mainly carry out less energy-intensive research activities.

11 Also, we cannot use interaction terms depending on the energy intensity of firms or a quantile regression approach, since under translog and linear logit cost functions, elasticities are a function of the estimated betas and the cost shares. These approaches might be suitable using simpler modeling techniques such as the Cobb-Douglas production function, which, however, is not suitable here as we show in “Data description and model selection” section.

12 Hence, firms do not change subsets over time. Additional subset-specific descriptive statistics of the factor cost shares are available in an online appendix.

13 Detailed test results can be found in an online appendix provided by the authors.

14 It is common practice to include a linear or a quadratic trend over time to increase the model fit.

15 Excluding the few observations that violate concavity, and re-estimating the two models does not affect our elasticity estimates.

16 Given the average energy cost share of 4%, an energy price increase of 1% raises the average firms’ costs by 0.04% *before* firms undertake any adjustments. A consequential change in the use of the factor capital of 0.09% *after* this moderate cost increase is substantial.

17 Remember that an unbalanced panel of firms is used. Hence, the first year of observation might differ for certain firms.

18 Detailed results are available in an online appendix. The similarity of results is due to the fact that energy cost shares of firms are relatively stable over time and thus most firms would be allocated to the same subset in any year they are observed.

19 If complementarity is stronger between energy and capital, capital use decreases more sharply following an energy price increase and thus, labor and material have to increase at a higher rate to hold output constant.

20 See Antimiani et al. (2016) for an assessment of the rate of carbon leakage and negative effects on competitiveness in the EU.
